# Novel Pan-Pim Kinase Inhibitors With Imidazopyridazine and Thiazolidinedione Structure Exert Potent Antitumor Activities

**DOI:** 10.3389/fphar.2021.672536

**Published:** 2021-05-03

**Authors:** Yuichi Sawaguchi, Ryuta Yamazaki, Yukiko Nishiyama, Masayuki Mae, Atsuhiro Abe, Hiroyuki Nishiyama, Fukiko Nishisaka, Tatsuya Ibuki, Toshio Sasai, Takeshi Matsuzaki

**Affiliations:** Yakult Central Institute, Yakult Honsha Co. Ltd., Tokyo, Japan

**Keywords:** pim kinase, imidazopyridazine, thiazolidinedione, pan-pim kinase inhibitor, anticancer drug, targeted therapy

## Abstract

Pim kinases are overexpressed in various types of hematological malignancies and solid carcinomas, and promote cell proliferation and survival. Here in this study, we investigated the preclinical profile of novel pan-Pim kinase inhibitors with imidazopyridazine and thiazolidinedione structure. Imidazopyridazine-thiazolidinediones inhibited activities of Pim kinases with IC_50_ values of tens to hundreds nanomolar. With YPC-21440 and/or YPC-21817, which exhibited especially high inhibitory activities against Pim kinases, we investigated *in vitro* and *in vivo* activities of imidazopyridazine-thiazolidinediones. *In silico* analysis of binding mode of YPC-21440 and Pim kinases revealed that it directly bound to ATP-binding pockets of Pim kinases. In the kinase panel tested, YPC-21440 and YPC-21817 were highly specific to Pim kinases. These compounds exerted antiproliferative activities against various cancer cell lines derived from hematological malignancies and solid carcinomas. Furthermore, they suppressed phosphorylation of Pim kinase substrates, arrested cell cycle at the G1 phase, and induced apoptosis in cultured cancer cells. In tumor xenograft models, YPC-21440 methanesulfonate and YPC-21817 methanesulfonate exerted antitumor activities. Furthermore, pharmacodynamic analysis with a xenograft model suggested that YPC-21817 methanesulfonate inhibited Pim kinases in tumors. In conclusion, our data revealed that imidazopyridazine-thiazolidinediones are novel Pim kinases inhibitors, effective on various types of cancer cell lines both *in vitro* and *in vivo*.

## Introduction

Pim kinases are serine/threonine kinases and composed of three isoforms, Pim-1, 2, and 3, which are highly homologous to each other. They are overexpressed in hematological malignancies, such as acute myeloid leukemia (AML), and solid tumors, such as prostate, gastric, and colon cancer, and their expression is correlated with poor prognosis in these types of cancers (Dhanasekaran et al., 2001; Popinanova et al., 2007; Peng et al., 2013; Lou et al., 2014; Cheng et al., 2017). In cancer signaling pathways, Pim-1, 2, and 3 are elucidated to have diverse functions. Pim-1, 2, and 3 directly phosphorylate and inhibit apoptotic protein Bad, and therefore contribute to cell survival and transformation (Yan et al., 2003; Aho et al., 2004; Li et al., 2006). Pim-2 is shown to phosphorylate eukaryotic initiation factor 4E binding protein (4EBP), a repressor of mRNA translation, and promote cell growth (Fox et al., 2003). Pim-1 promotes degradation of p21^Cip1/WAF1^, a negative regulator of the cell cycle, by direct phosphorylation and was suggested to progress cell cycle from the G1 to S phase (Banerjee et al., 2014). Furthermore, Pim-1 has functional roles in chemoresistance, and Pim-1, 2, and 3 contribute to tumor immune evasion (Chen et al., 2009; Weirauch et al., 2013; Szydłowski et al., 2017; Daenthanasanmak et al., 2018). Moreover, lack of significant toxicity in Pim-1, 2, and 3 triple-knockout mice suggests that their signaling network is not essential for functions of normal tissues (Mikkers et al., 2004). These reports imply that Pim kinase inhibitors are expected not only to exhibit cytotoxicity specifically in cancer cells, but to contribute to overcoming chemoresistance and immunotherapy. Additionally, Pim kinases are indicated to be complementary to each other in c-Myc-induced mice lymphoma models (van der Lugt et al., 1995; Mikkers et al., 2002), suggesting that activities of all Pim kinase isoforms should be inhibited in order to exert anticancer effects on cancers in which Pim kinase signaling contributes to malignancy.

Previously, we found that a pan-Pim kinase inhibitor, compound **2** with rhodanine-benzoimidazole structure, suppressed proliferations of both hematological and solid cancer cell lines *in vitro* (Sawaguchi et al., 2017). However, this inhibitor showed little activity against human cancer xenografts, probably due to its poor metabolic stability (data not shown). In the present study, through the optimization of compound **2** aiming to improve its metabolic stability, we discovered imidazopyridazine-thiazolidinediones and evaluated their inhibitory effects against Pim-1, 2, and 3 kinases. Moreover, with YPC-21440 and YPC-21817, which inhibited Pim kinase activities most potently among the test compounds, we demonstrated that imidazopyridazine-thiazolidinediones exerted anticancer effects against various types of cancer cell lines both *in vitro* and *in vivo*.

## Materials and Methods

### Materials

(Z)-5-([3-{4-(4-methylpiperazin-1-yl)phenyl}imidazo[1,2-*b*]pyridazin-6-yl]methylene)thiazolidine-2,4-dione (YPC-21440), (Z)-5-([3-{4-(4-pentylpiperazin-1-yl)-3-fluorophenyl}imidazo[1,2-*b*]pyridazin-6-yl]methylene)thiazolidine-2,4-dione (YPC-21813), (Z)-5-([3-{4-(4-decylpiperazin-1-yl)-3-fluorophenyl}imidazo[1,2-*b*]pyridazin-6-yl]methylene)thiazolidine-2,4-dione (YPC-21814), (Z)-5-([3-{4-(4-ethylpiperazin-1-yl)-3-fluorophenyl}imidazo[1,2-*b*]pyridazin-6-yl]methylene)thiazolidine-2,4-dione (YPC-21817), (Z)-5-([3-{3-(tert-butyl)-4-(4-methylpiperazin-1-yl)phenyl}imidazo[1,2-*b*]pyridazin-6-yl]methylene)thiazolidine-2,4-dione (YPC-21867), (Z)-5-([3-{4-(4-methylpiperazin-1-yl)phenyl}imidazo[1,2-*b*]pyridazin-6-yl]methylene)thiazolidine-2,4-dione methanesulfonate (YPC-21440 MsOH), and (Z)-5-([3-{4-(4-ethylpiperazin-1-yl)-3-fluorophenyl}imidazo[1,2-*b*]pyridazin-6-yl]methylene)thiazolidine-2,4-dione methanesulfonate (YPC-21817 MsOH) were chemically synthesized by Yakult Honsha (Tokyo Japan). Test compounds were dissolved in dimethyl sulfoxide (DMSO) or 5% glucose, for experiments *in vitro* or *in vivo*, respectively.

### Antibodies

The following antibodies were used for immunoblotting: Anti-phospho-Bad (pSer112) (#SAB4300050, Sigma-Aldrich; Merck KgaA, Darmstadt, Germany), Anti-BAD antibody produced in rabbit (#SAB3500336, Sigma-Aldrich; Merck KgaA), Phospho-4E-BP1 (Ser65) (#9451, Cell Signaling Technology, Danvers, MA, USA), 4E-BP1 antibody (#9452, Cell Signaling Technology), p-p21 (Thr145)-R Antibody (#sc-20220-R, Santa Cruz Biotechnology, Santa Cruz, CA, United States), p21 Antibody (C-19) (#sc-397, Santa Cruz Biotechnology), Monoclonal Anti-α-tubulin produced in mouse (#T9026, Sigma-Aldrich; Merck KgaA), and Monoclonal Anti-β-Actin antibody produced in mouse (#A5316, Sigma-Aldrich; Merck KgaA).

### *In Vitro* Pim Kinase Activity Assay

The inhibition ratios of test compounds for Pim-1, 2 (Life Technologies; Thermo Fisher Scientific Inc., MA, United States), 3 (Merck KgaA), and FLT3 (Life Technologies; Thermo Fisher Scientific Inc., MA, United States) were determined using a modified FRET-based Z′-LYTE kinase assay kit (Invitrogen; Thermo Fisher Scientific Inc.). The Ser/Thr 7 peptide and Tyr-2 peptide were used as substrates for Pim kinases and FLT3, respectively. The reactions were carried out in a 384-well plate with a 10 µL reaction volume per well containing 1 µM substrates in 50 mM HEPES buffer with pH 7.5, 0.01% BRIJ-35, 10 mM MgCl_2_, 1 mM EGTA, and an appropriate amount of kinases (Pim-1: 0.03 μg/ml, Pim-2: 0.2 μg/ml, Pim-3: 0.2 μg/ml, FLT3: 0.1 μg/ml) with test compounds. The final reaction concentrations of ATP were 400, 5, 100, and 500 µM for Pim-1, 2, 3, and FLT3 respectively. After 1 h incubation, reactions were developed and terminated, and fluorescence ratios were calculated following the manufacturer’s protocol.

### Binding Detection Based on SPR Platform

The interactions between test compounds and Pim-1 were detected by surface plasmon resonance platform BIACORE T200 (GE Healthcare Life Sciences, Buckinghamshire, England). Pim-1 was diluted to 20 μg/ml in 10 mM acetate buffer (pH 5.0), and then immobilized as a ligand in the N-hydroxysuccinimide/ethyl 3-(3-dimethylaminopropyl) carbodiimide-pre-activated CM5 sensor chip, following blocking by ethanolamine. Test compounds were diluted at 6.5, 13, 25, or 50 µM in a vehicle of 5% DMSO (v/v) in phosphate buffered saline (PBS). The dilutions were injected as analyte flow liquid phase with PBS containing 5% DMSO (v/v) as running buffer at a constant flow rate of 30 μL/min. A 2-min association time was set. The data were collected by Biacore Control Software. Kinetics and affinity parameters were evaluated with the Langmuir model (1:1) using BIA evaluation software.

### Docking Analysis of YPC-21440 to Pim-1

Docking analysis was performed using MOE software (MOE ver.2018, Chemical Computing Group Inc., Montreal, Canada). The three-dimensional structure of Pim-1 was obtained from the protein data bank (PDB code 3F2A). The Pim-1 structure was hydrogenated using the Protonate 3D module. After partial charges were assigned using Merck Molecular Force Field 94x (MMFF94X) (Halgren, 1996), hydrogen atoms were minimized. The Alpha Site Finder module was used for definition of a ligand-binding site targeting the ATP binding site of Pim-1. In the docking analysis using the MOE-dock module, YPC-21440 conformation previously generated by the stochastic search method was posed on the binding site. The docked poses were scored by the London dG scoring function. The top 30 poses were further optimized by the GBVI/WSA dG scoring function with the Generalized Born/Volume Integral solvation model (Labute, 2008), and the top 5 poses were finally output.

### Kinetic Assay

Enzyme kinetic experiments were performed at pH 7.5 in 50 mM HEPES buffer with 1 mM EGTA, 10 mM MgCl_2_, and 0.01% Brij-35. Reactions were assembled in 384-well plate wells by adding 20 ng/ml of Pim-1, 100 ng/ml of Pim-2, or 10 ng/ml of Pim-3 into separate reaction mixtures containing 1 μM fluorescently labeled Caliper peptide substrate (FL-peptide 20, PerkinElmer Inc., Waltham, MA, United States) with various concentrations of ATP and YPC-21440. The final ATP concentrations varied from 31.3 to 1000 μM and YPC-21440 varied from 0 to 1 μM. Plates were immediately placed into a Caliper LabChip EZ Reader II (PerkinElmer Inc.) and wells were sampled periodically throughout an 1-hour reaction period for initial reaction rate. The fluorescent product and substrate were separated and monitored on a Caliper microfluidic instrument. The conversion of substrate was calculated with Caliper software. Ki values were determined from the double reciprocal Lineweaver-Burk plot.

### Kinase Selectivity Assay

Kinase selectivity was evaluated against a panel of 46 protein tyrosine kinases and serine/threonine kinases at Carna Biosciences (Hyogo, Japan). Test compounds were diluted with assay buffer (20 mM HEPES, pH7.5, 0.01% Triton X-100, 2 mM DTT) and added to 384-well plates. Peptide substrates and ATP in assay buffer were added to the wells. After that, each of the kinases prepared in assay buffer was also added to the wells and mixed to start the reaction. After a period of incubation at room temperature, the reaction was stopped by Termination Buffer (QuickScout Screening Assist MSA; Carna Biosciences). Finally, the plate was put on a LabChip 3000 system (Caliper Life Sciences, Hopkinton, MA, United States) and a droplet of the reaction mixture was applied for electrophoretic separation in the chips of the machine. The enzyme conversion data were then used for analysis.

### Cells and Cell Cultures

We obtained colon cancer HCT116 and HT-29 cells, prostate cancer PC-3, DU145, and LNCaP cells, lung cancer NCI-H460 and NCI-H522 cells, breast cancer MDA-MB-231 cells, AML MV-4-11 cells, and chronic myeloid leukemia (CML) K-562, MEG-01, and KU812 cells from American Type Culture Collection (Manassas, VA, United States); stomach cancer MKN45 cells from the Health Science Research Resources Bank (Osaka, Japan); pancreatic cancer AsPC-1 and MIA PaCa-2, and lung cancer A549 cells from DS Pharma Biomedical (Osaka, Japan). All cell lines were cultured in RPMI1640 medium (Life Technologies; Thermo Fisher Scientific Inc.) with 10% (v/v) fetal bovine serum (FBS; Sigma-Aldrich; Merck KgaA) at 37°C in 5% CO_2_.

### Cytotoxicity Assay for Cell Survival

Cells were seeded in 96-well plates and test compounds were added to the cultures at several concentrations. After 96 hours, cell viabilities were measured with WST-8 and the concentrations of test compounds equivalent to the IC_50_ values of cell viabilities were calculated.

### Immunoblot Analysis

Cells were plated overnight and then treated with test compounds for 4 hours. Then, the cells were lyzed in solubilization buffer [10 mM Tris-HCl (pH7.4), 0.1% (w/v) NP-40, 0.1% (w/v) sodium deoxycholate, 0.1% (w/v) SDS, 0.15 M NaCl, 1 mM EDTA, 10 µM aprotinin] with phosphatase inhibitor cocktails (Nakarai Tesque, Kyoto, Japan), and subjected to SDS-PAGE using 15% (w/v) gels under reducing conditions. The separated proteins were electrotransferred to Immobilon transfer membranes (Merck Millipore, Darmstadt, Germany). Then, each membrane was reacted with primary antibodies, which were subsequently complexed with appropriate horseradish peroxidase-conjugated secondary antibodies. Signals were detected using the ECL Western Blotting Detection System (GE Healthcare Life Sciences) and protein expression was quantified with a LAS-3000 Luminescent Image Analyzer (GE Healthcare Life Sciences). The protein bands were quantified using ImageQuant TL software (GE Healthcare Life Sciences).

### Cell Cycle Analysis

Cells were treated with YPC-21440 for 24 h. After treatment, cells were fixed and stained with propidium iodide using the Cell Cycle Phase Determination kit (Cayman Chemical, Ann Arbor, MI, United States) and analyzed by Guava EasyCyteTM Plus System (Merck Millipore).

### Apoptosis Assay

For caspase-3/7 activity assay, the Caspase-Glo 3/7 Assay System (Promega, Madison, WI) was used according to the manufacturer’s protocol. Cells were grown in 96-well plates and treated with YPC-21440 for 24 h. The Caspase-Glo 3/7 reagent was then added to each well and incubated for 1 h, and caspase-3/7 activity was measured. For evaluation of nuclear morphology, cells were pre-cultured in 1% FBS medium for 3 h, and then treated with YPC-21440 for 48 h. After treatment, cells were stained in 0.5 μg/ml Hoechst 33342 for 30 min. Subsequently, cell morphology was observed and images were captured from random visual fields using fluorescence microscopy (Biozero BZ-8100, Keyence, Osaka, Japan). For the Cell Death ELISA Assay, cells were treated with YPC-21440 for 24 h in 1% FBS medium. After treatment, DNA fragmentation was detected using the Cell Death Detection ELISA^PLUS^ (Roche Applied Science, Mannheim, Germany).

### *In Vivo* Xenograft Study

All experiments with animals were conducted in accordance with the Guidelines of the Yakult Central Institute and protocols approved by the Animal Experimental Committee of the Yakult Central Institute.

For evaluation of antitumor activity, MV-4-11 (5 × 10^6^ cells) or PC-3 cells (2 × 10^6^ cells) were injected subcutaneously in the inguinal region of 6-week-old male BALB/cSlc-*nu/nu* mice (Japan SLC, Inc., Shizuoka, Japan), with Matrigel (BD Biosciences, San Jose, CA, USA). When the average tumor volume (length × width^2^ × 1/2) had reached 40 to 120 mm^3^, animals were randomized into groups of seven mice for vehicle control group of MV-4-11 xenograft model or five mice for the other groups (day 1). Test compounds were injected into the tail vein every 4 days from day 1, with an administration volume of 10 ml/kg. Tumor volumes were measured 2 times per week and body weights of the mice were monitored on 2 or 3 times per week as an indicator of tolerability. On day 22 or 16, antitumor activity was evaluated by weighing tumor tissues. Tumor growth inhibition, TGI (%), was calculated according to formula (1 − T/C) × 100, where T and C are the mean tumor weights for the experimental and vehicle control groups, respectively. The differences between the mean tumor weights for comparing groups were analyzed using Dunnett’s test, where *P* < 0.05 was considered to be significant.

For the pharmacodynamics study, mice with PC-3 tumors were treated with vehicle or YPC-21817 MsOH, with 5 mice per time point. Tumors were resected 1, 2, 6, or 24 h after injection and lyzed for immunoblot analysis.

### Statistical Analysis

Statistical analyses were performed using Dunnett’s test. *p*-values < 0.05 were considered significant.

## Results

### Inhibition of Kinases by Imidazopyridazine-Thiazolidinediones and Their Mechanisms of Action

**FIGURE 1:**
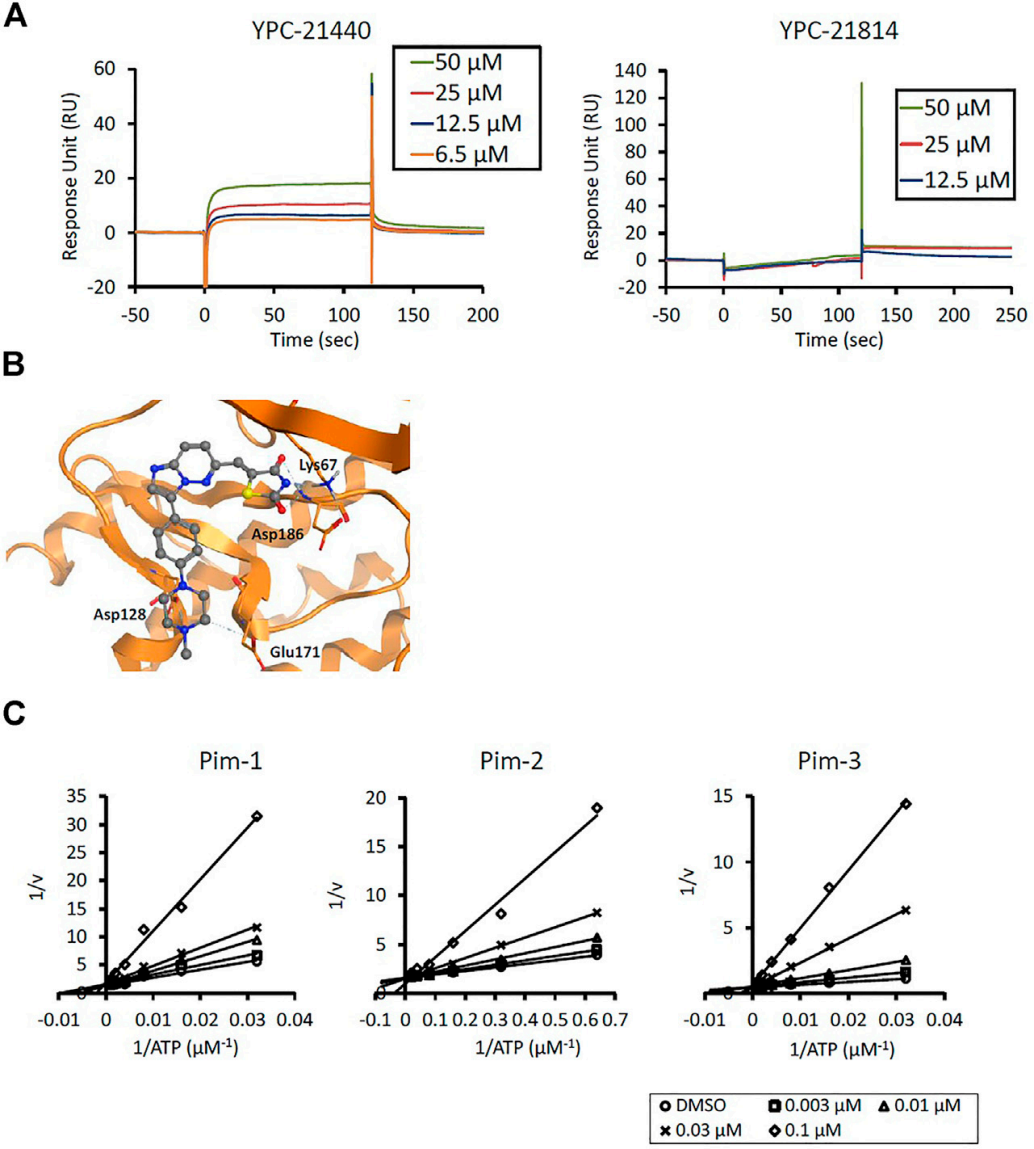
Mechanisms of inhibition of Pim kinases by YPC-21440 **(A)** Binding affinities of YPC-21440 and YPC-21814 to Pim-1 determined by SPR. Real-time measurements of the interactions of test compounds with Pim-1 using the BIACORE T200 instrument are shown. The curves represent the interaction of various concentrations of test compounds with the protein. **(B)** Docking model of YPC-21440 to the ATP binding site of Pim-1. YPC-21440 is shown in a ball-and-stick representation. The hydrogen bonds between YPC-21440 and Pim-1 residues are indicated with broken lines. **(C)** Lineweaver-Burk analysis of the effects of YPC-21440 on Pim-1, 2, and 3 activities. Pim-1, 2, and 3 assays were performed at serial diluted concentrations of ATP and the reciprocal values are plotted. Pim-1, 2, and 3 activities were determined either in the presence or absence of 0.003, 0.01, 0.03, or 0.1 μM YPC-21440.

**FIGURE 2:**
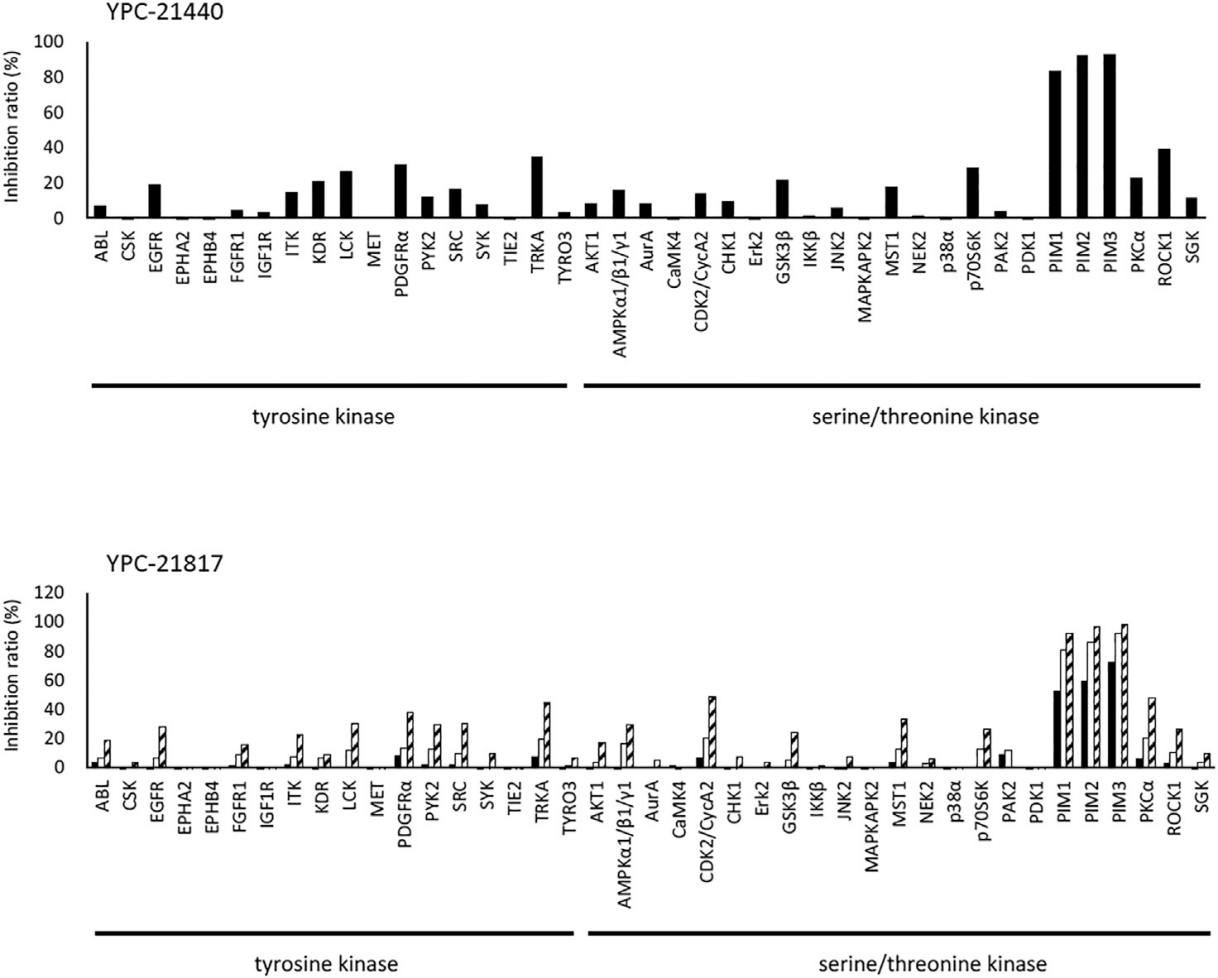
Kinase inhibition selectivity profile of YPC-21440 and YPC-21817. The inhibitory ratio of YPC-21440 at 0.3 μM and YPC-21817 at 0.03, 0.1, and 0.3 μM for 19 kinds of tyrosine kinases and 27 kinds of serine/threonine kinases. Percentage of kinase inhibition is shown in the bar graph.

We previously synthesized a pan-Pim kinases inhibitor, compound **2** with rhodanine-benzoimidazole structure (Supplementary Figure S1) (Sawaguchi et al., 2017). We optimized this inhibitor and identified several imidazopyridazine-thiazolidinediones (Table 1). Among these compounds, YPC-21440 and YPC-21817 most potently inhibited Pim-1, 2, and 3, with IC_50_ values ranging from 0.012 to 0.11 μM. Hereafter, with these two compounds as representatives, we evaluated the pharmacological actions of imidazopyridazine-thiazolidinediones. In order to clarify the inhibitory mechanisms of imidazopyridazine-thiazolidinediones against Pim kinases, we analyzed interactions between YPC-21440 and Pim kinases (Figure 1). Biacore analysis revealed that YPC-21440 bound Pim-1 in a concentration-dependent manner (Figure 1A). In addition, YPC-21814, which had no effects on Pim kinase activities, did not bind to Pim-1. In *silico* docking analysis, it was found that the thiazolidinedione and methylpiperazine group of YPC-21440 may interact with Lys67/Asp186 and Asp128/Glu171 in Pim-1, respectively, through hydrogen bonds (Figure 1B). Furthermore, in the kinetic assay with Pim kinases, the Lineweaver-Burk lines of each concentration of YPC-21440 crossed on the vertical axis, which demonstrated that YPC-21440 inhibited Pim kinases in an ATP competitive manner (Figure 1C). Next, to investigate the specificity of imidazopyridazine-thiazolidinediones, we examined the inhibitory effects of YPC-21440 and YPC-21817 against tyrosine and serine/threonine kinase panels (Figure 2). Both of these compounds at 3 μM inhibited Pim kinases most potently in the panels. Moreover, at 0.03 and 0.1 µM YPC-21817, more apparent differences were observed between the inhibitory effects against Pim kinases and the other kinases, compared to that at 0.3 µM. Additionally, we assessed the inhibitory effects against FLT3 kinase, which is frequently activated in acute myeloid leukemia. The IC_50_ values of YPC-21440 and YPC-21817 were 0.39 and 2.0 µM, respectively.

**TABLE 1:** Effect of substitution on R_1_ position of imidazopyridazine.

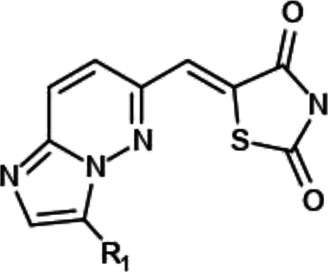				
Compound name	R_1_	IC_50_ values for enzyme activities (µM)		
		Pim-1	Pim-2	Pim-3
YPC-21440	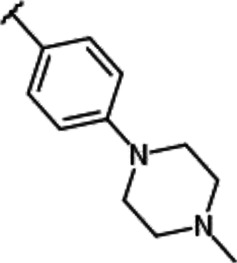	0.095	0.012	0.016
YPC-21813	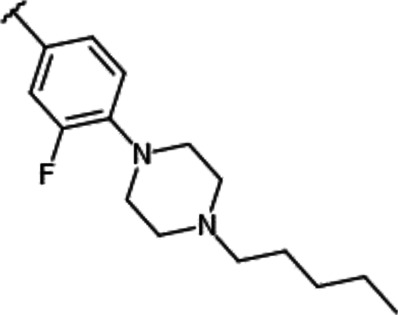	0.43	0.49	0.36
YPC-21814	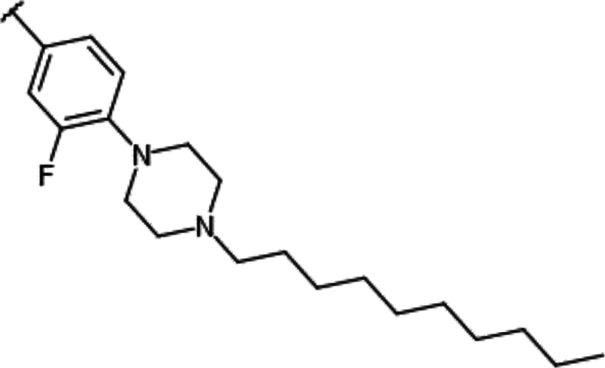	> 1.8	> 1.8	> 1.8
YPC-21817	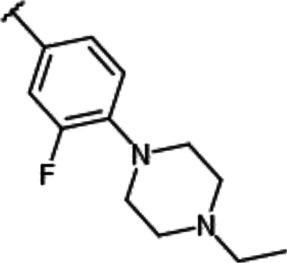	0.11	0.039	0.063
YPC-21867	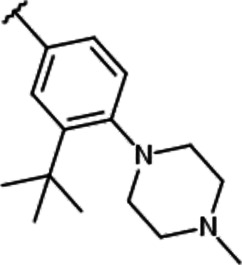	0.12	0.19	0.54

### Cytotoxicities and Cellular Activities of Imidazopyridazine-Thiazolidinediones

**FIGURE 3:**
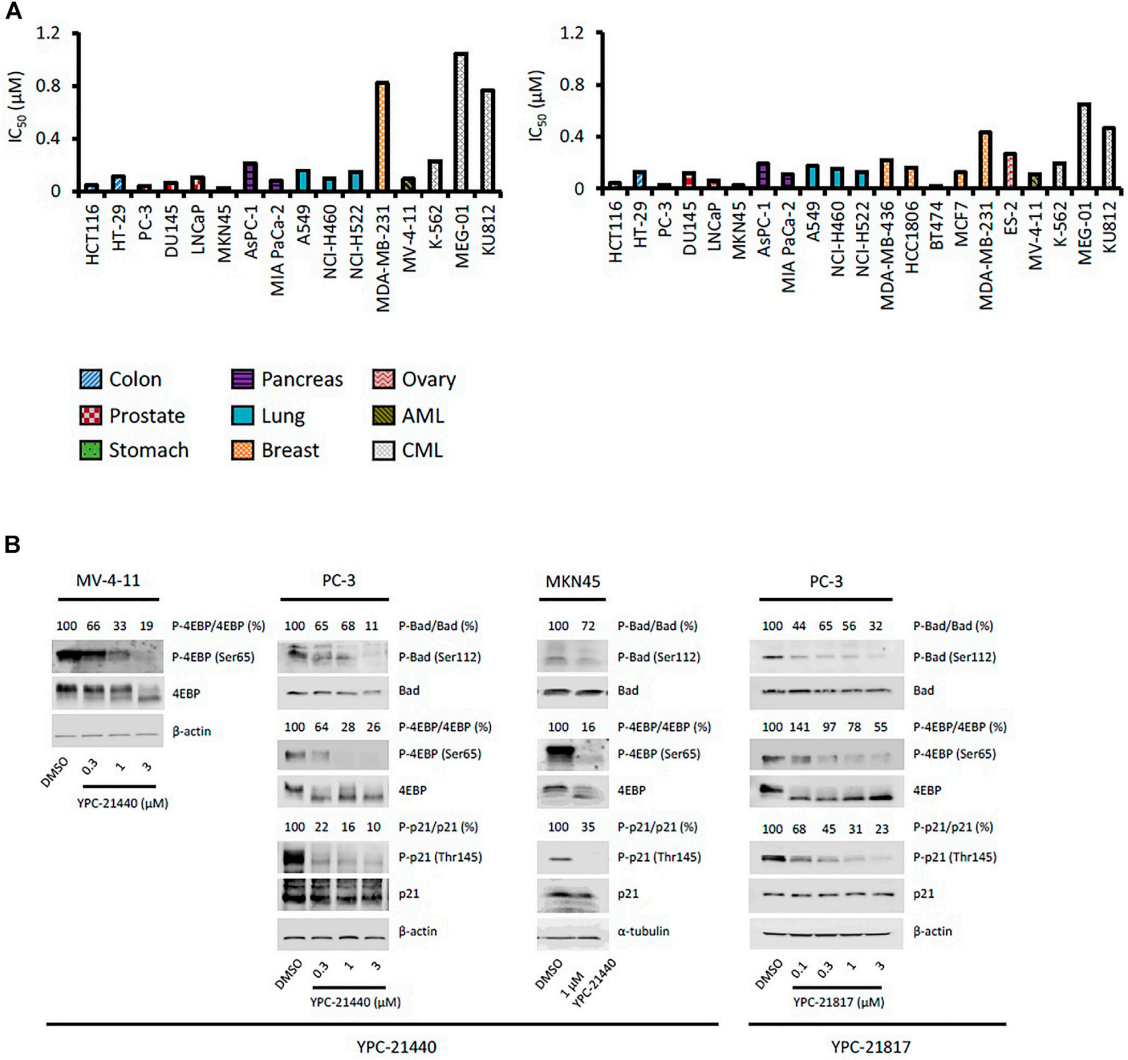
Effects of YPC-21440 and YPC-21817 on cell proliferation and phosphorylation of Pim kinases substrates in cultured cancer cell lines. **(A)** Inhibitory effects of YPC-21440 and YPC-21817 on cancer cell proliferation. The indicated cell lines were treated with test compounds and cell viability was analyzed by MTT assay. The results are indicated as IC_50_ values. **(B)** Immunoblot analysis of phosphorylation on Pim kinases substrates in cancer cell lines treated with YPC-21440 and YPC-21817. The indicated cell lines were treated with DMSO alone or test compounds for 4 h, then harvested and lyzed. The expressions of phosho-4E-BP (Thr37/46), total 4E-BP, phospho-Bad (Ser112), total Bad, phosoho-p21 (Thr145), and total p21 were analyzed by immunoblot. Beta-actin or α-tubulin was used as a loading control. Densitometry of Western blotting of phospho-4EBP/4EBP, phosho-Bad/Bad, and phopho-p21/p21 were analyzed relative to that in control cells.

**FIGURE 4:**
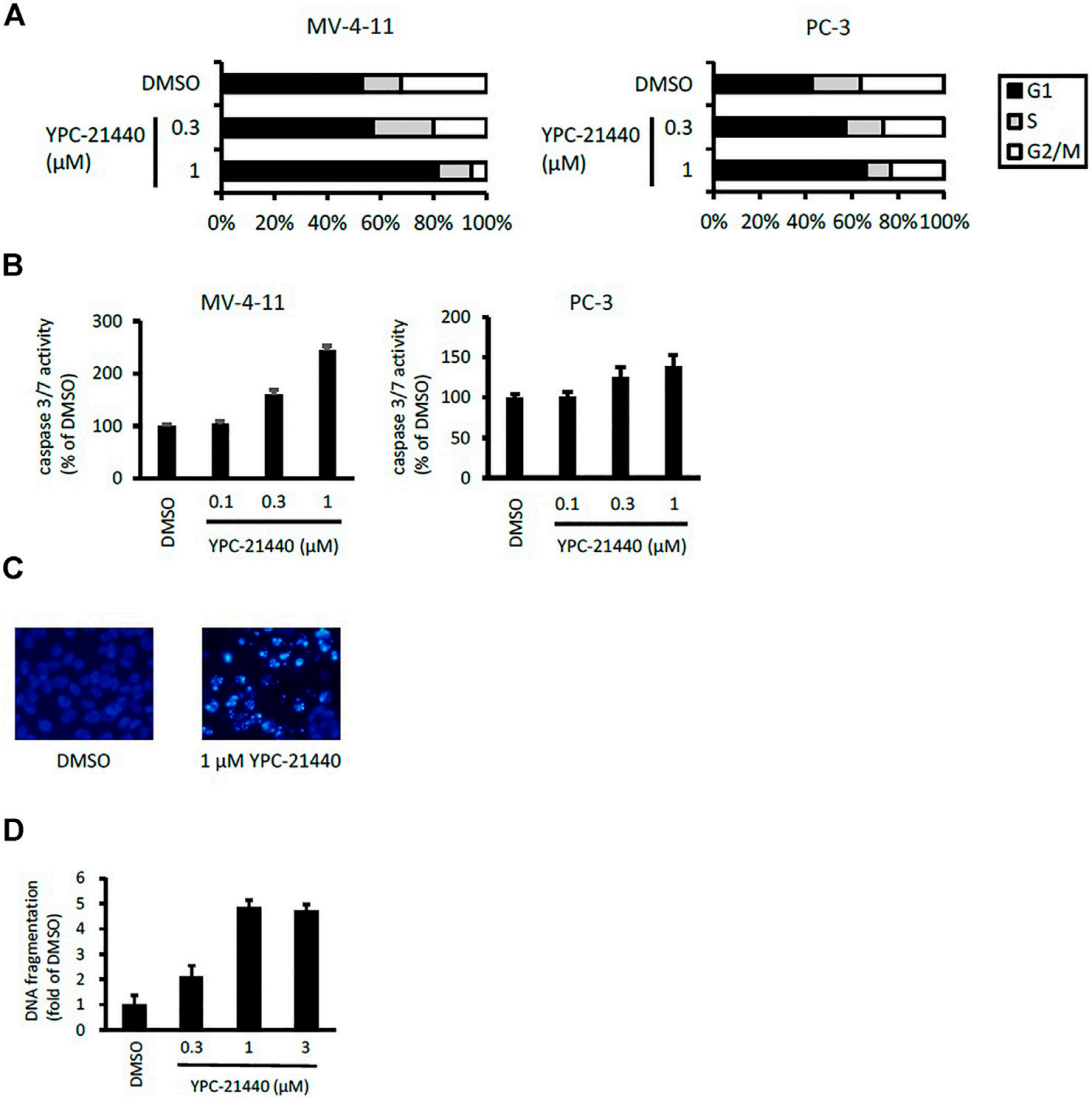
Cell cycle distribution and apoptosis of cancer cell lines after treatment with YPC-21440. **(A)** Effects of YPC-21440 on cell cycle progression. MV-4-11 and PC-3 cells were incubated with DMSO or YPC-21440 for 24 h. Then, cell cycle was analyzed by flow cytometry after propidium iodide staining. **(B–D)** Evaluation of apoptosis in cancer cell lines treated with YPC-21440. Caspase 3/7 activities in MV-4-11 and PC-3 cells were measured after treatment of DMSO or YPC-21440 for 24 h **(B)**. HCT116 cells were treated with DMSO or YPC-21440 for 48 h, and then stained by Hoechst 33342. Apoptotic cells showed strong fluorescent signals (bright turquoize color) outlining the chromatin-condensed nuclei, whereas normal cells appeared weakly stained **(C)**. DNA fragmentation in HCT116 cells was quantified using an ELISA kit after treatment of DMSO or YPC-21440 for 24 h **(D)**.

Using YPC-21440 and YPC-21817, we evaluated activities of imidazopyridazine-thiazolidinediones in cancer cell lines (Figure 3). Compound **2** suppressed proliferation of MV-4-11 and PC-3 (Sawaguchi et al., 2017), which suggested that imidazopyridazine-thiazolidinediones may also suppress proliferation of hematological and solid cancer cells. As expected, YPC-21440 and YPC-21817 inhibited proliferation of a broad spectrum of cancer cell lines, with IC_50_ values of 0.027 to 1.0 µM and 0.024 to 0.65 µM, respectively (Figure 3A). In addition, MV-4-11 among the hematological cancer cell lines, and HCT116, PC-3, and MKN45 among the solid cancer cell lines exhibited especially high sensitivities to both compounds, and therefore, these cell lines were chosen to investigate cellular activities of YPC-21440 and YPC-21817. In order to assess the inhibitory activities of YPC-21440 and YPC-21817 against Pim kinases in cells, we determined the phosphorylation levels of substrates of Pim kinases, Bad, 4EBP, and p21, in cancer cell lines treated with these compounds (Figure 3B). The phosphorylation level of 4EBP was decreased in MV-4-11 treated with YPC-21440, although that of Bad and p21 was not detected in this cell line. Moreover, the phosphorylation levels of Bad, 4EBP, and p21 were decreased in PC-3 and MKN45 treated with YPC-21440, and in PC-3 treated with YPC-21817. To assess the effect on cell cycle progression, MV-4-11 and PC-3 were incubated with YPC-21440 and the cell cycles were analyzed (Figure 4A). The percentages of cells in G1 phase after treatment of DMSO, 0.3, or 1 µM YPC-21440 was 52, 55, and 77% in MV-4-11; and 41, 55, and 65% in PC-3, respectively. These results indicated that YPC-21440 arrested cell cycle at the G1 phase in both cell lines. By using caspase 3/7 activities, chromatin condensation, and DNA fragmentation as indexes, we explored apoptosis induction of YPC-21440 (Figure 4B–D). YPC-21440 at 0.3 and 1 µM increased caspase 3/7 activities in MV-4-11 and PC-3. Furthermore, in HCT116, YPC-21440 induced chromatin condensation at 1 µM and DNA fragmentation at ≥ 0.3 µM. These data suggested that YPC-21440 induced apoptosis in cancer cells.

### *In vivo* Antitumor Activities of Imidazopyridazine-Thiazolidinediones

**FIGURE 5:**
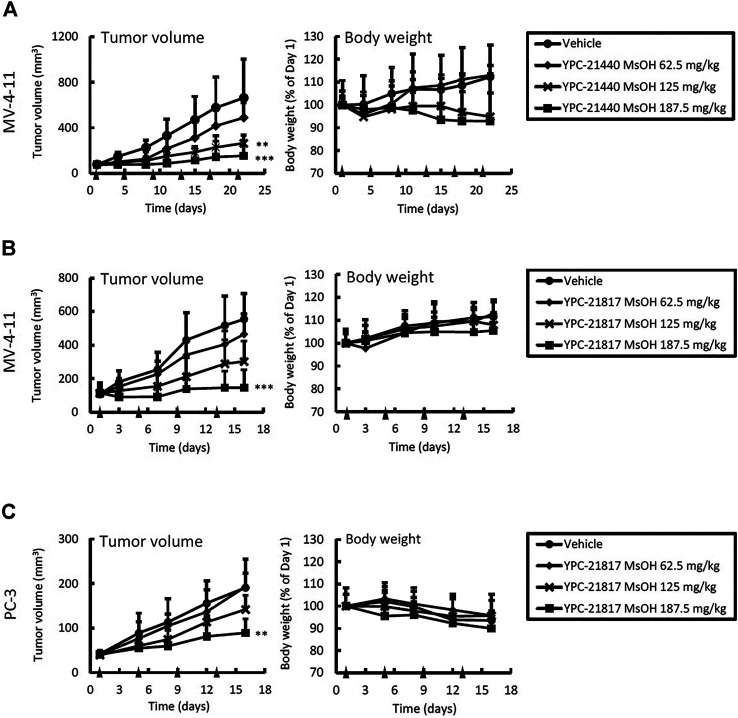
Antitumor activities and tolerability of YPC-21440 MsOH and YPC-21817 MsOH in xenograft model mice of cancer cell lines. MV-4-11 **(A, B)**, and PC-3 **(C)** tumor xenograft nude mice were randomized into vehicle or treatment groups (*n* = 5/group) at day 1 and received YPC-21440 MsOH **(A)** and YPC-21817 MsOH **(B, C)**. Tumor volume, tumor weight, and body weight were measured as described in Materials and Methods. Data are presented as means ± SD. Statistical analysis was conducted on tumor weight. **P < 0.01, ***P < 0.001.

Given the potent *in vitro* cytotoxicities of imidazopyridazine-thiazolidinediones against MV-4-11 and PC-3, we examined *in vivo* antitumor activities of imidazopyridazine-thiazolidinediones in xenograft models of these cell lines. YPC-21440 MsOH and YPC-21817 MsOH were administered intravenously to xenograft models. In the MV-4-11 xenograft model, TGI of YPC-21440 MsOH at 62.5, 125, and 187.5 mg/kg/dose were 26 (not significant vs. vehicle), 60 (*P* < 0.01 vs. vehicle), and 77% (*P* < 0.01 vs. vehicle), respectively (Figure 5A). During the evaluation period, body weight decreased as much as 5.2 ± 10 and 7.1 ± 21% compared with day 1 in groups of YPC-21440 MsOH at 125 and 187.5 mg/kg/dose, respectively. TGI of YPC-21817 MsOH at 62.5, 125, and 187.5 mg/kg/dose were, respectively, 20 (not significant vs. vehicle), 39 (not significant vs. vehicle), and 70% (*P* < 0.001 vs. vehicle) in the MV-4-11 xenograft model, and 0.92 (not significant vs. vehicle), 8.4 (not significant vs. vehicle) and 48% (*P* < 0.001 vs. vehicle) in the PC-3 xenograft model (Figure 5B,C). Serious decrease in body weight was not observed in either of the xenograft models treated with YPC-21817 MsOH during the evaluation period.

### Pharmacodynamic Activity of Imidazopyridazine-Thiazolidinediones

**FIGURE 6:**
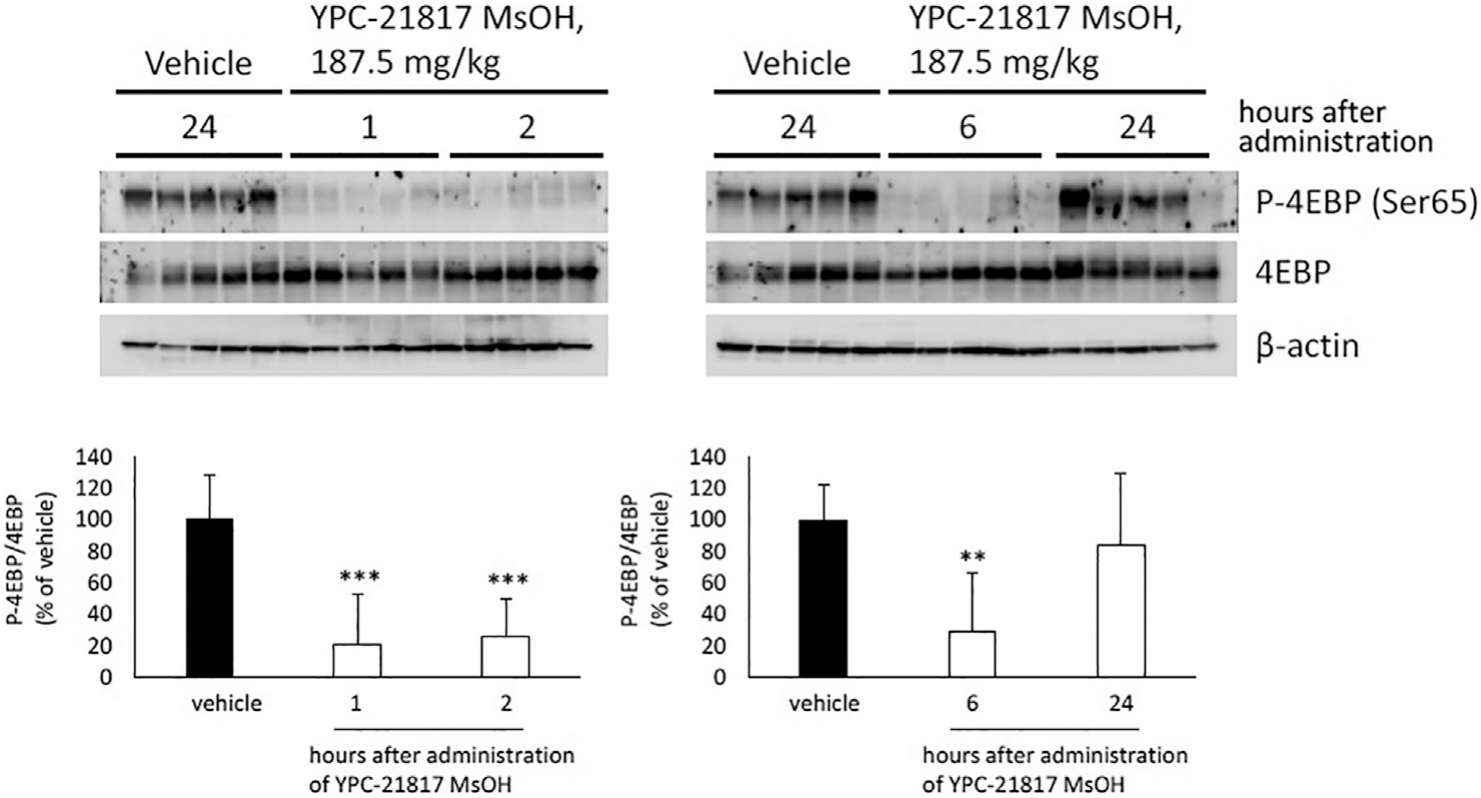
Pharmacodynamic analysis of YPC-21817 MsOH in PC-3 xenograft model mice. Mice with PC-3 tumor received vehicle or 187.5 mg/kg of YPC-21817 MsOH. One, 2, 6, or 24 h after injection, tumors were resected. **(A)** The expressions of phospho-4EBP and 4EBP in the tumors were analyzed by immunoblot. **(B)** Densitometry of Western blotting of phospho-4EBP/4EBP. Graphs represent mean per group (*n* = 5 per group). Data are presented as means ± SD. ***P < 0.001, **P < 0.01 between vehicle and YPC-21817 MsOH-treated groups.

Given that imidazopyridazine-thiazolidinediones exerted antitumor activity in the PC-3 xenograft model, we evaluated the pharmacodynamic activity of imidazopyridazine-thiazolidinediones with this model treated with YPC-21817 MsOH. Following a single dose of YPC-21817 MsOH at 187.5 mg/kg, the phosphorylation level of 4E-BP in tumors was analyzed at indicated time points to assess the inhibitory effect against Pim kinases (Figure 6). The phosphorylation level of 4E-BP was signigficantly decreased at 1, 2, and 6 hours after compound administration, and then almost completely recovered 24 hours after compound administration compared to earlier time points. Thus, YPC-21817 may inhibit Pim kinases in xenograft tumor tissues.

## Discussion

In the present report, we identified imidazopyridazine-thiazolidinediones as novel Pim kinase inhibitors, and assessed their antitumor activities in preclinical models of hematological malignancies and solid tumors.

Among imidazopyridazine-thiazolidinediones, YPC-21440 and YPC-21817 exerted especially potent inhibitory activities against all isoforms of Pim kinases. Surface plasmon analysis, Lineweaver-Burk plot analysis, and docking analysis with YPC-21440 suggested that imidazopyridazine-thiazolidinediones interacted with ATP-binding pockets of Pim kinases to inhibit their kinase activities. In addition, the docking model demonstrated that there was a hydrophilic region beyond the methyl group on the piperazine ring of YPC-21440 (data not shown). Therefore, it was presumed that YPC-21814 did not bind to Pim-1 because YPC-21814 had a longer hydrocarbon chain on the piperazine ring than YPC-21440. We previously synthesized compound **2** with rhodanine and benzoimidazole structure, analogous to imidazopyridazine and thiazolidinedione structure, respectively (Sawaguchi et al., 2017). YPC-21440 interacted with Pim-1 *in silico* with the binding pose similar to compound **2**, which specifically inhibited Pim kinases. This result implied that imidazopyridazine-thiazolidinediones were also specific to Pim kinases. As expected, YPC-21440 and YPC-21817 exhibited high specificity for Pim kinases in the kinase panel assessed in this study. In cultured cancer cells derived from hematological malignancies and solid tumors in which Pim kinases were considered to promote the progression, YPC-21440 and YPC-21817 displayed antiproliferative activities. The acute myeloid leukemia cell line MV-4-11, one of the highly sensitive cells for both compounds, harbors the constitutively active FLT3 kinase mutant (Quentmeier et al., 2003). Furthermore, some Pim kinase inhibitors also inhibit FLT3 kinase and exhibit strong cytotoxicity against AML cell lines (Chen et al., 2011; Czardybon et al., 2018). These reports led us to evaluate the effects of YPC-21440 and YPC-21817 on FLT3 kinase activity. Although both compounds inhibited FLT3 kinase activity, the IC_50_ values for FLT3 kinase were several to several tens of times higher than those for Pim kinases. Therefore, it was suggested that both compounds suppressed proliferation of MV-4-11 mainly by Pim kinase inhibition. YPC-21440 and YPC-21817 decreased the phosphorylation of Pim kinase substrates at the micromolar range in cancer cells. In the same concentration range, YPC-21440 arrested cell cycle at the G1 phase and induced apoptosis. These results suggested that imidazopyridazine-thiazolidinediones inhibited the activities of Pim kinases in cells and consequently prevented biological events concerning signaling of Pim kinases.

YPC-21440 MsOH demonstrated a significant antitumor effect compared with the vehicle control group in the dose range that did not induce serious weight loss in the MV-4-11 xenograft model. Although the antitumor effect of YPC-21440 MsOH was evaluated in the PC-3 xenograft model, the experiment was not completed due to serious body weight loss in YPC-21440 MsOH-treated groups, which was probably induced by both administration of the compound and tumor growth (data not shown). However tumor growth tended to be suppressed by administration of YPC-21440 MsOH in the PC-3 xenograft model, which suggested that YPC-21440 MsOH potentially also exhibited antitumor effects in this model. In both the MV-4-11 and PC-3 xenograft models, YPC-21817 MsOH demonstrated a significant antitumor effect compared with the vehicle control group with little effect on body weight. Furthermore, pharmacodynamic analysis of PC-3 xenografts dosed with YPC-21817 MsOH support that it suppressed Pim kinase activities in tumors. These results suggest that imidazopyridazine-thiazolidinediones exhibit potent *in vivo* activities.

The present results revealed that imidazopyridazine-thiazolidinediones potently inhibit Pim kinases and demonstrated antitumor activities against hematological and solid cancer cell lines. On the other hand, from the proliferation assay with LGB321 (a selective inhibitor of Pim kinases) against a large panel of cancer cell lines, it was suggested that cell proliferation of solid tumors was less dependent on Pim kinases compared to that of hematological malignancies (Garcia et al., 2014). This observation implies that imidazopyridazine-thiazolidinediones have inhibitory activities against oncogenic kinases not involved in our kinase panel, because they exhibited broad-spectrum cytotoxicity not only in hematological but in solid cancer cells. For example, a previous report described that the PI_3_K/Akt/mTOR pathway, which partly shared signaling factors (PRAS40, Bad, etc.) with Pim kinase pathways, contributes to resistance to inhibition of Pim kinases (Rebello et al., 2018). In other reports, compounds with imidazopyridazine structure were shown to inhibit PI_3_K activity and thereby exert antitumor activity (Stec et al., 2011; Mevellec et al., 2018). These findings suggest that the cytotoxicity of imidazopyridazine-thiazolidinediones in various cancer cell lines is explained at least partly by their inhibitory effects against PI_3_K kinase. Additional work is needed in the future to confirm the kinase specificity of imidazopyridazine-thiazolidinediones.

To date, several clinical studies of Pim kinase inhibitors, including SGI-1776, AZD1208, and PIM447, have been performed. The studies of SGI-1776 and AZD1208 were terminated owing to their dose-limiting toxicity and the lack of clinical response, respectively (NCT00848601, NCT01489722). Furthermore, although PIM447 displayed therapeutic effects on multiple myeloma patients as a single agent in a phase I study, its treatment outcome has not been reported in other types of carcinoma (Raab et al., 2019). In the present study, we have demonstrated that imidazopyridazine-thiazolidinediones exhibited antitumor activities against hematological and solid cancer cell lines both *in vitro* and *in vivo*. Hence, imidazopyridazine-thiazolidinediones are expected to be developed as novel Pim kinase inhibitors effective on various types of cancers.

## Data Availability

The raw data supporting the conclusion of this article will be made available by the authors, without undue reservation.
