# Supplementary figures and images for: Novel Pan-Pim Kinase Inhibitors With Imidazopyridazine and Thiazolidinedione Structure Exert Potent Antitumor Activities
Source: Front Pharmacol. 2021 May 3;12:672536. doi: 10.3389/fphar.2021.672536 (PMC8126654; doi:10.3389/fphar.2021.672536)

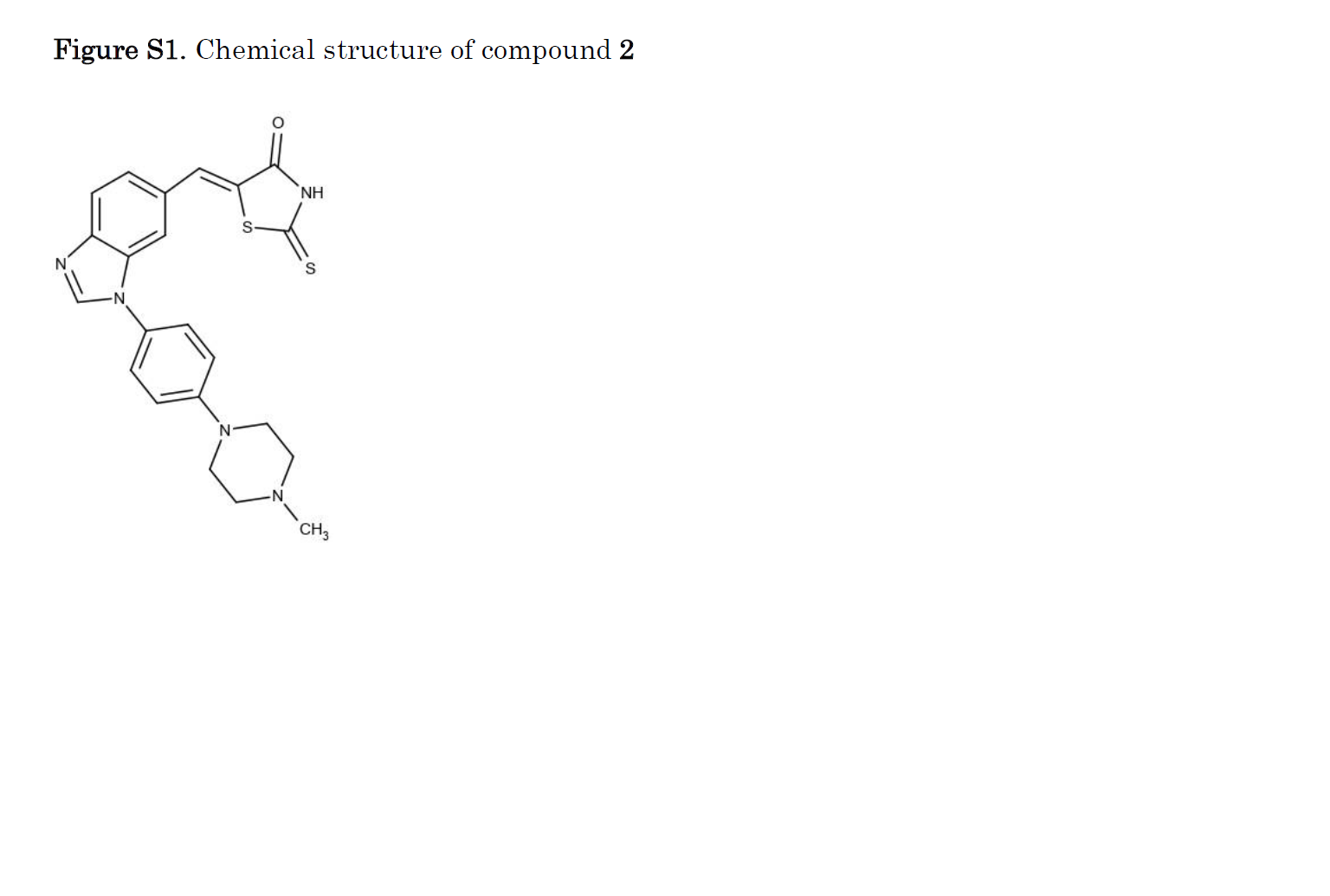

Supplement: Supplementary file 1 [file Image1.TIF]
